# Recurrent Pneumonia in Children: A Reasoned Diagnostic Approach and a Single Centre Experience

**DOI:** 10.3390/ijms18020296

**Published:** 2017-01-29

**Authors:** Silvia Montella, Adele Corcione, Francesca Santamaria

**Affiliations:** Department of Translational Medical Sciences, Federico II University, Via Pansini 5, 80131 Naples, Italy; amina2004@virgilio.it (S.M.); dedeee@hotmail.it (A.C.)

**Keywords:** community-acquired pneumonia, lower respiratory tract infections, recurrent pneumonia, diagnosis, children

## Abstract

Recurrent pneumonia (RP), i.e., at least two episodes of pneumonia in one year or three episodes ever with intercritical radiographic clearing of densities, occurs in 7.7%–9% of children with community-acquired pneumonia. In RP, the challenge is to discriminate between children with self-limiting or minor problems, that do not require a diagnostic work-up, and those with an underlying disease. The aim of the current review is to discuss a reasoned diagnostic approach to RP in childhood. Particular emphasis has been placed on which children should undergo a diagnostic work-up and which tests should be performed. A pediatric case series is also presented, in order to document a single centre experience of RP. A management algorithm for the approach to children with RP, based on the evidence from a literature review, is proposed. Like all algorithms, it is not meant to replace clinical judgment, but it should drive physicians to adopt a systematic approach to pediatric RP and provide a useful guide to the clinician.

## 1. Introduction

Community-acquired pneumonia (CAP) is a major cause of morbidity and mortality in developing and developed countries [1]. Approximately 6% of infants experience at least one episode of pneumonia during the first two years of life [2]. Recurrent pneumonia (RP) is defined as at least two episodes of pneumonia in one year or three episodes ever, with intercritical radiographic clearing of densities [3]. Incidence data indicate that RP occurs in 7.7%–9% of all children with CAP [4,5,6,7,8,9]. As a result, RP represents a frequent presenting manifestation in the general pediatric practice and is a very common reason for referral to pediatric chest physicians [4].

Data on the underlying causes of RP in children come from retrospective case series [4,5,6,7,8,9,10], and prospective studies are lacking. The methodology for prospective studies of RP implies the recruitment of a large cohort of children, a thorough investigation of the cases, and the generation of predictive indices for confirming or excluding the diagnosis. Eventually, the validity and reliability of the indices should be determined before they are definitely applied to the general population. The challenge for the physician approaching RP is to discriminate between children with self-limiting or minor problems, that do not require a diagnostic work-up, and those with an underlying disease, for whom further investigation is mandatory.

The aim of the current review is to discuss a reasoned approach to the diagnosis of RP that helps physicians to decide when the time has come to move from the decision of not investigating further, to the determination that a work-up is mandatory. We carried out an electronic keyword literature search for English articles published on this topic up until 13 December 2016, using the Scopus, Web of Science, PubMed, and MEDLINE databases. We excluded studies conducted exclusively on adults, but included those with a study population comprising children (or adolescents) and adults. The terms “diagnosis” AND (community-acquired pneumonia OR pneumonia OR lower respiratory tract infection OR recurrent pneumonia) were used as keywords in combination with each other, and the studies identified were evaluated for the selection of relevant literature. In addition, a manual search was conducted to evaluate references contained in the review articles. This literature review prompted us to develop a diagnostic algorithm for the evaluation of RP. A pediatric case series is also presented, in order to document a single centre experience of RP.

## 2. Which Children Should Undergo a Diagnostic Work-Up?

Healthy children, particularly of a preschool age, are at risk of pneumonia during autumn and winter, when multiple respiratory viruses spread [2,11]. When children start daycare, there may be a substantial increase in the number of airway infections that they are exposed to, which may become recurrent. In the evaluation of children with RP, the first step is to distinguish between otherwise healthy “but unlucky” subjects, and those with an underlying disorder that requires further investigation [12]. A thorough diagnostic work-up is not required when: infections are self-limiting; other organs or systems are not involved; there are relatively long periods of clinical wellness, at least during the summer; the child has normal growth and a normal physical examination; the family history for genetic or infectious respiratory disorder is negative; there is a quick response to treatment and a complete recovery after the episode [12,13,14,15,16,17,18]. The presence of any risk factor for RP should be ruled out by a careful recording of the clinical history (Table 1), as cases presenting risk factors may experience earlier and more severe episodes.

Conversely, children with early symptoms and/or occasional need for hospitalization, those with short intercritical periods of wellness, and most of all, those who present risk factors for RP may require a wait-and-see approach for a period of 6 to 12 months [14]. As many risk factors as possible should be removed before deciding to perform any additional diagnostic test. If the frequency and characteristics of pneumonia do not change during the follow-up period, despite the removal of avoidable risk factors, further investigations are needed.

Several signs in the medical history or the physical examination suggest the presence of an underlying disease in a child with RP, and thus should lead to further investigation (Table 2).

Additional investigations are mandatory if RP starts early, severe symptoms and signs and/or any systemic involvement or serious local complications are reported, or when unusual causative pathogens are isolated [1]. Likewise, diagnostic testing is strongly recommended if any relative has a genetic disorder that primarily affects the respiratory tract, or there is an unexplained death from infection in the family.

It is well known that radiologic abnormalities of pneumonia may require even longer than 12 weeks before the clearing of densities occurs [30,31]. Therefore, determining which child should be investigated relies on clinical judgment, that should take into account the patient’s history, the clinical course of the episode, and any symptoms and/or signs indicating the presence of an underlying disease.

## 3. Which Underlying Diseases Should Be Suspected in Children with RP?

In one of the first studies on the etiology of RP, Owayed et al. [5] found that most of the children hospitalized during the first episode of pneumonia had a known predisposing condition for pneumonia recurrence, including neuromotor disorders with feeding problems, gastroesophageal reflux, or congenital heart disease. Other conditions, such as asthma, airway malacia, and vascular malformations, do not usually present with RP, and whether their coexistence in a child represents a causal association, or merely coincides by chance, is not always clear [32].

Differential diagnosis of RP in the same area is different from RP that affects different or multiple areas of the lung (Table 3 and Table 4).

**Figure 1:**
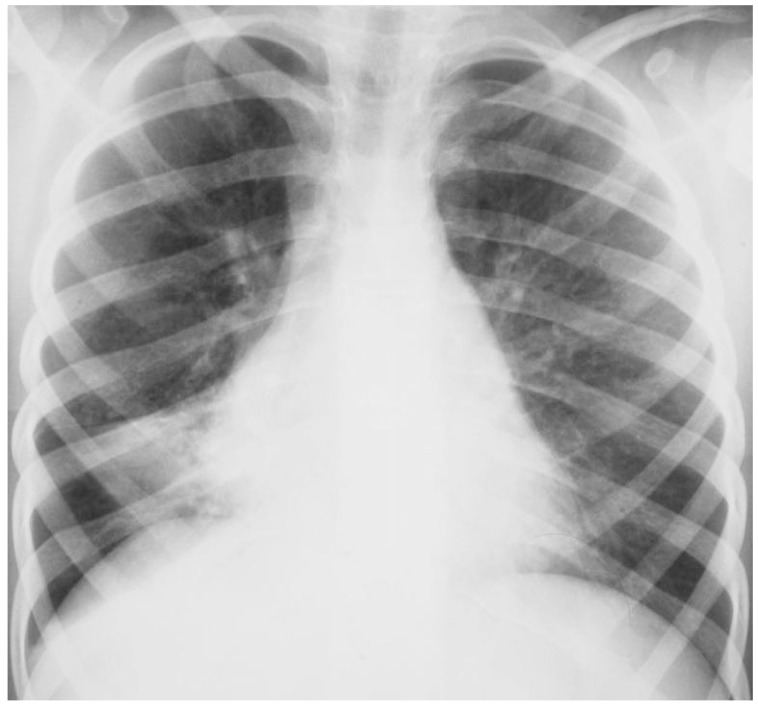
Chest radiography of a 7-year-old girl with middle lobe syndrome.

However, exceptions are possible, and thus, physicians should take into account that this criterion is a rule of thumb and is not extrapolated from an evidence-based clinical guideline. Middle lobe syndrome is the most frequent cause of focal RP in the clinical practice [33]. This is due, at least in part, to the fact that the middle lobe bronchus is relatively narrow and long, and arises from the bronchus intermedius with an acute angle (Figure 1).

Moreover, it can be compressed by enlarged adjacent lymphnodes. Finally, no collateral ventilation is present between the middle lobe and other lobes, and this decreases the likelihood of reinflation after the development of atelectasis. When densities recur in the same area, localized intraluminal obstruction, focal extraluminal compression, or congenital airway abnormalities, should be suspected. In children, the most frequent cause of intraluminal obstruction is a retained foreign body [33]. This should be suspected in the presence of sudden-onset dyspnea, cough and RP, especially if the clinical history is positive for choking episodes. However, there may be no definite history, and this can significantly delay the diagnosis. Extraluminal compression can be caused by enlarged lymphnodes, and/or enlarged or aberrant vessels. Structural airway anomalies include focal bronchomalacia or bronchial stenosis, and localized bronchiectasis. Pulmonary sequestration, cystic adenomatoid malformation, and bronchogenic cysts represent the most frequent causes of bronchopulmonary malformations which may cause RP. Although rare in childhood, endobronchial tumours (i.e., bronchial carcinoid, mucoepidermoid tumours, hemangiomas, papillomas, leiomyomas, and mucous gland tumours) may result in RP [34]. Finally, tuberculosis infection is re-emerging as a cause of severe pneumonia, that might also recur [14].

A cluster of genetic disorders that primarily involve the respiratory tract and the host’s defense against pathogens, i.e., cystic fibrosis (CF), primary ciliary dyskinesia (PCD), and primary immune defects (ID), may present with RP in different areas. There are several reasons why pediatricians should be confident in the approach to genetic RP. First, recognition of the underlying disorder is crucial, in order to refer the patient to a specialized centre where a multidisciplinary approach can help to reduce morbidity. Second, early and adequate treatment might also include innovative or last generation therapies. Finally, genetic counseling is mandatory for sustaining both patients and their relatives in the family planning.

When RP is associated with chronic wet cough, pancreatic insufficiency, rectal prolapse, electrolyte disorders, liver disease, or nasal polyps, CF should be excluded by sweat chloride determination and/or genetic analysis [35]. Features of PCD include neonatal respiratory distress, abnormal situs, rhinorrhea since the newborn period, serous otitis media with hearing loss, chronic wet cough due to recurrent airway infections, and a positive family history for PCD [15]. The presence of serious infections in other organs or systems (e.g., skin or gastrointestinal tract), or of pneumonia sustained by atypical bacteria or presenting with massive infection, especially if with a prolonged clinical course, dictates that ID should be ruled out [16,36]. In particular, antibody deficiencies, complement disorders, and neutrophil abnormalities should be suspected and excluded as early as possible, as their early identification and management may help to preserve lung function and growth [36]. As international travel is common, a detailed history of where the child has been is crucial. A medication history is also important and should take into account self-prescriptions [36,37]. A clinical clue to RP associated with pulmonary hemorrhagic syndromes is persistent iron deficiency anemia, as the child may swallow rather than expectorate blood [38]. Children with chronic neurological disorders frequently have respiratory symptoms and signs [39]. Causative mechanisms include recurrent aspiration, secondary to gastroesophageal reflux and/or uncoordinated swallowing, and reduced mucociliary clearance, due to hypotonia and an impaired cough reflex that may be worsened by chronic administration of anti-epileptic drugs [40,41]. The act of coughing is a crucial defensive mechanism, and entails the presence of efficient muscular strength, especially by expiratory muscles. Therefore, conditions affecting muscle strength lead to recurrent infections, mucus plugging, and atelectasis [39]. Moreover, chronic aspiration may result in the progressive loss of the cough reflex. Therefore, a careful history relating to choking should be recorded, and neurological incoordinate swallowing should be excluded. Finally, obesity, a well recognized global epidemic also in children, increases the risk of aspiration pneumonia that, if recurs, may result in respiratory failure [42]. Nevertheless, even after taking into account all of the possible underlying diseases, in a proportion of children the etiology of RP may remain obscure.

## 4. Which Diagnostic Approach to Pediatric RP?

**Figure 2:**
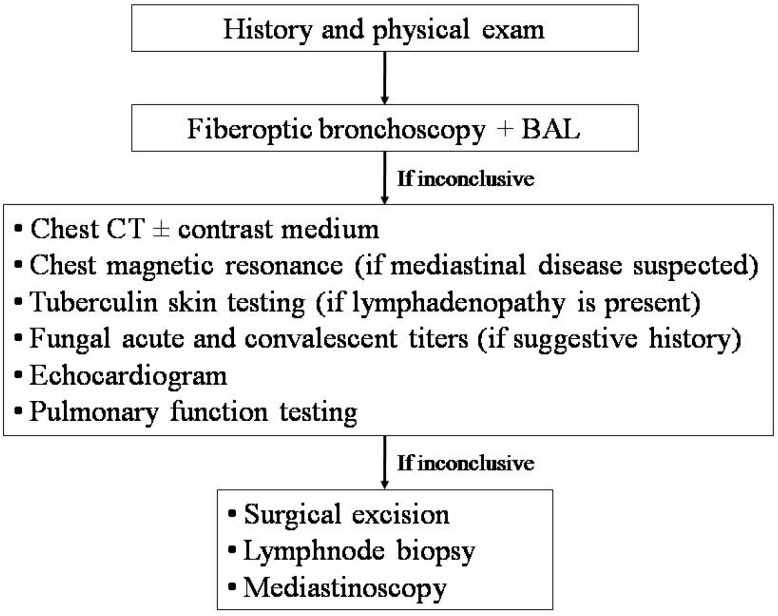
Diagnostic approach to children with recurrent pneumonia in the same area. BAL, bronchoalveolar lavage; CT, computed tomography.

There is no evidence base to inform the clinician of the optimal timing of investigations or the optimal sequence of the diagnostic work-up in children with RP. However, if the same area is involved, or if localized rales and rhonchi are detected, even in intercritical periods, crucial diagnostic tests are represented by fiberoptic bronchoscopy, to exclude focal airway disease (i.e., foreign bodies, intraluminal obstructions, mucus plugs, or extrinsic compressions), and chest high resolution computed tomography (HRCT), to rule out focal parenchymal disease (Figure 2) [12,13,14,33,36].

During bronchoscopy, samples from airways or alveolar spaces should be obtained for culture and cytologic examination. HRCT should be performed when the child has no acute infection, in order to avoid issues in the interpretation of the scans. If possible, this should be compared with previous imaging to check whether the anomaly is new. Administration of an intravenous medium should be considered, in order to obtain a detailed picture of the vascular anatomy at CT. A congenital pulmonary malformation is likely if the abnormality drains into the systemic circulation or has an arterial supply from the aorta. If mediastinum or chest wall disease is suspected, or in cases presenting with soft-tissue masses, chest magnetic resonance is extremely effective [43]. The lack of radiation exposure makes this imaging technique very attractive, and therefore it has also been introduced in clinical practice for obtaining anatomical and functional information on children with recurrent or chronic lung disease [44,45,46]. Unfortunately, the need for general anesthesia in young children might represent a limitation to its use, unless essential [46]. Tuberculin skin testing, and acute and convalescent titers for coccidioidomycosis, blastomycosis, and histoplasmosis, should be performed if specific pointers are present [33]. An echocardiogram is essential for investigating an enlarged cardiac chamber which is compressing the bronchus.

Many children are too young for pulmonary function testing (PFT), although techniques that measure the airway resistance or the lung clearance index are applicable in the preschool-age [47,48,49,50]. Much useful information can be obtained from spirometry, in addition to lung volumes and carbon monoxide transfer determination, if the child is able to perform these tests. However, PFT only provides a global evaluation of lung function, contributes no clues to the specific contribution of each lung or lobe, and is not sensitive enough to distal airway disease [51,52].

If the diagnostic work-up in cases with RP in the same area is inconclusive, a more invasive approach, including surgical excision, lymphnode biopsy, and mediastinoscopy, may be indicated [36]. Finally, the possibility that RP in the same area may be the first presentation of a generalized illness should always be taken into account.

**Figure 3:**
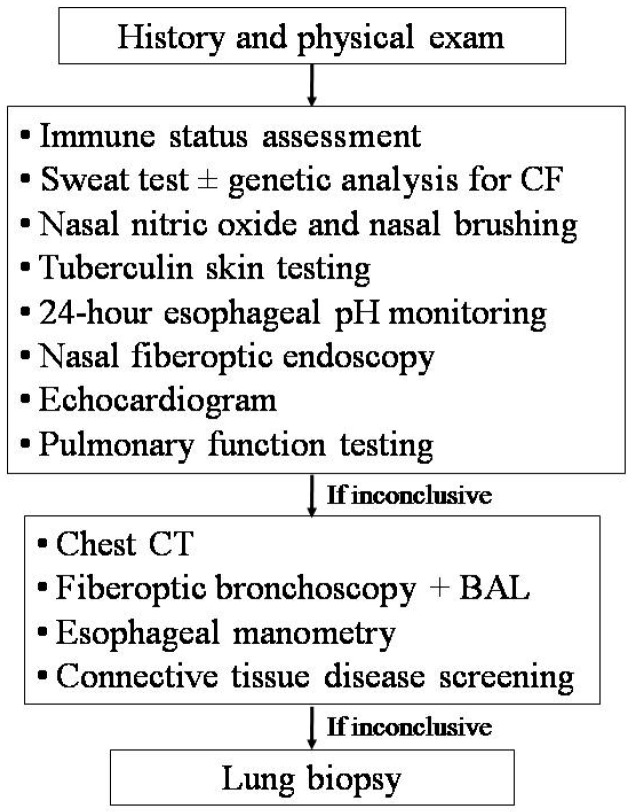
Diagnostic work-up for children with recurrent pneumonia in different areas. CF, cystic fibrosis; CT, computed tomography; BAL, bronchoalveolar lavage.

The differential diagnosis of RP involving different areas of the lung is wide, and consequently, the number of possible tests is great. Therefore, a targeted approach based on suspicion, driven by the clinical history and physical exam, is strongly recommended (Figure 3) [12,13,14,33,36].

Ideally, the diagnosis should be confirmed or excluded with the minimum possible number of the least-invasive confirmatory tests. Patients should undergo a blood test to assess their immune status (i.e., blood cell counts, serum immunoglobulins, total IgE, IgG sub-classes, lymphocyte sub-populations, C3, C4, CH50, and specific antibody responses to tetanus toxoid, capsular polysaccharides of *Haemophilus influenzae* type B, *Streptococcus pneumoniae*, and/or measles, rubella, mumps, and hepatitis B viruses), nasal fiberoptic endoscopy, sweat test and/or genetic analysis for CF, 24-h esophageal pH monitoring, tuberculosis screening, nasal nitric oxide measurement, and ciliary motility/ultrastucture study [13,14,53]. Some children with RP have normal levels of IgG, but low levels of IgG subclasses. Indeed, IgG2 subclass deficiency is associated with poor antibody responses to polysaccharides. If all of these investigations are negative but RP persists, bronchoscopy with bronchoalveolar lavage (BAL) is recommended, in order to identify potentially untreated pathogens, perform a cytologic examination, and assess the lipid burden in alveolar macrophages (an indirect sign of pulmonary aspiration) [14]. However, the use of lipid-laden macrophages measured on BAL lacks specificity [54]. Therefore, if lipid-laden macrophages are not found in the BAL, significant aspiration is unlikely. The assessment of BAL pepsin has a higher specificity [55], but this test can only be performed in a few centers. A tube esophagram is the usual investigation performed if late-presenting congenital H-type fistula is suspected, but a normal test does not exclude this diagnosis, and direct visualization with bronchoscopy may be needed [36,56]. Sophisticated manometry should be considered if esophageal dysmotility defects are suspected. A pulmonary hemorrhage can be diagnosed in the presence of iron-laden macrophages at BAL. However, this test can not discriminate among the different causes of bleeding. If there is no evidence of an underlying predisposing disease, the condition is usually considered to be idiopathic pulmonary hemosiderosis, and a confirmatory lung biopsy is not required [36]. However, an open lung biopsy should be considered in the presence of recurrent bleeding, in order to exclude neutrophilic capillaritis [57]. If a connective tissue disease is suspected, screening tests include the erythrocyte sedimentation rate, circulating immune complexes, complement studies, rheumatoid factor, anti-neutrophil cytoplasmic antibodies, double-stranded DNA, and anti-glomerular basement membrane antibody (for children with pulmonary hemorrhage and renal disease).

In our own series of 113 children with RP radiologically confirmed during each episode, we applied the thorough diagnostic work-up proposed above and found an underlying disease in 83% of the cases (Table 5).

**Figure 4:**
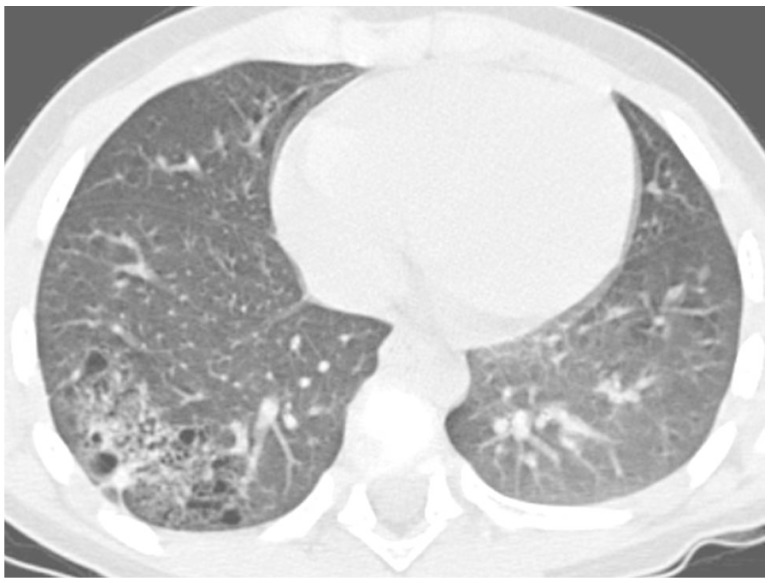
High resolution computed tomography scan of a 6-year-old boy with recurrent pneumonia in the right lower lobe due to congenital cystic adenomatoid malformation (diagnosis confirmed at lung biopsy after lobectomy).

The most frequent causes of RP in the same area were right middle lobe syndrome and congenital malformations, including pulmonary sequestration and congenital cystic adenomatoid malformation (Figure 4).

The most common underlying conditions in children with RP in different areas (75% of the whole study population) were genetic diseases (i.e., ID, CF, and PCD), and this is probably the reason why the age at the first episode of pneumonia was significantly lower in these patients. Findings from this study population indicate that both groups of subjects have a similar prevalence of risk factors for RP.

Compared to previous studies on the causes of RP [5,6,7,8,9,10], our series shows some similarities and differences (Table 6).

As in almost all previous reports, our cases were diagnosed at a tertiary care centre, and the rate of diagnosis of any underlying disease was quite similar. The frequency of immune defects, heart/vessel anomalies, CF, and tuberculosis was not different from what has been reported by other authors. Conversely, we found a higher prevalence of lung/airway diseases and PCD, and a lower frequency of recurrent aspiration and/or gastroesophageal reflux. These differences might be primarily explained by the fact that the diagnostic approach to children with RP varies according to the clinician’s experience and the patient’s clinical course. The diagnostic reasoning is obviously influenced by the physician’s habits, preferences, and convictions [58,59,60]. Consequently, both the type and the extent of investigation performed for RP will have an impact on the ultimate diagnosis. Second, when an underlying cause is identified, it is generally considered a sufficient explanation for RP. Therefore, further investigations are not pursued. Third, the natural history of RP in children plays a key role in the choice to investigate further. For example, if blood tests reveal no abnormalities, a more invasive work-up (i.e., 24-h esophageal pH monitoring or bronchoscopy with BAL) is only carried out if the child continues to experience episodes of pneumonia [13]. This might explain the relatively low proportion of recurrent aspiration and/or GER in our series, when compared to previous reports. Finally, some tests are not available in all centres. This might be the reason why we had a higher prevalence of PCD compared to studies performed many years ago [5,8], or in developing countries [6,7].

## 5. Conclusions

Recurrent pneumonia is still a diagnostic challenge in pediatrics. Many children with RP do not need a thorough investigation, as pneumonia episodes are not frequent and/or severe, or they stop recurring over time. Determining which case should be investigated relies on clinical judgment, depending on a careful history and physical examination, whether the child is improving clinically, and whether there is any feature suggestive of an underlying condition. Early treatment of the child’s underlying condition is crucial in order to stabilize lung disease and thus prevent progressive pulmonary function deterioration [61].

Regrettably, the lack of prospective studies of RP makes it difficult to develop clinical guidelines that specifically recommend practice in children with RP. A huge range of tests is available and there is no evidence base to guide the clinician on the most appropriate timing or sequence of investigations. Ideally, the diagnosis should be confirmed or excluded with the minimum number of the least-invasive tests. The economic burden of an extensive diagnostic work-up should always be kept in mind. Generally, RP in the same location is mainly due to middle lobe syndrome, localized airway obstruction/compression, or parenchimal disease, while RP affecting different/multiple sites is associated with systemic disorders. A management algorithm for the approach to children with RP, based on the evidence from a literature review, is hereby proposed. Like all algorithms, it is not meant to replace clinical judgment, but it should drive physicians to adopt a systematic approach to pediatric RP.

## Figures and Tables

**Table 1:** Risk factors for recurrent pneumonia in children.

Condition	Proposed Underlying Mechanisms
Prematurity/bronchopulmonary dysplasia [19,20]	Inadequate immunity due to low maternal antibodies levels
	Impaired lung function
	Altered innate immunoregulatory response of the lungs to respiratory pathogens secondary to neonatal hyperoxia
Atopy [21,22]	Defective innate immune response of epithelial cells
	Interleukin 13-dependant reduced mucociliary clearance
Tobacco smoke exposure [23,24,25,26]	Neonatal low lung volume and impaired toll-like receptor-mediated immune response
	Suppressed phagocytic activity of neutrophils and monocytes/macrophage cells secondary to reduced production of oxygen radicals
	Increased bacterial adherence
	Impaired lung function
Over-crowding [27]	Increased exposure to respiratory pathogens
Indoor and outdoor pollution [28,29]	Distal bronchial and alveolar inflammation

**Table 2:** Pointers that should lead to further investigation in a child with recurrent pneumonia.

**Pointers in the History**	- Unexplained death, severe infections or multisystem disease in the family
	- Unusual organisms or any feature of a systemic immunodeficiency
	- Respiratory infection *plus* extrapulmonary infections or other disease
	- Chronic rhinosinusitis and/or otitis media from the first months of age
	- Very sudden onset of symptoms
	- Chronic moist cough/sputum production
	- More severe symptoms or irritability after feeds and when lying down
	- Continuous, unremitting, or worsening symptoms
**Pointers in the Physical Examination**	- Severe infection
	- Persistent infection and failure of expected recovery
	- Prolonged interstitial pneumonia with no detectable infective cause
	- Digital clubbing, signs of weight loss, failure to thrive
	- Unusually severe chest deformity
	- Fixed monophonic wheeze or asymmetric wheeze
	- Signs of cardiac or systemic disease
	- Persistence of lung crackles on auscultation for more than eight weeks

**Table 3:** Underlying causes of recurrent or chronic pneumonia in the same lobe or segment.

Right Middle Lobe Syndrome
Localized airway obstruction
Endobronchial foreign body
Localized malacia or bronchiectasis
Congenital malformation; congenital webs; complete cartilage rings
Mucus plug
Carcinoid or other pedunculated tumor; intramural airway tumor
Inflammatory pseudotumor secondary to previous intubation
Localized airway compression
Vascular ring; pulmonary artery sling
Enlarged lymphnodes
Enlarged cardiac chamber due to right-to-left shunting; cardiomyopathy
Fibrosing mediastinitis
Mediastinal cancer
Parenchimal disease
Congenital malformation
Infection in residual cystic change after a cavitating pneumonia or tuberculosis
Lung cancer

**Table 4:** Underlying causes of recurrent pneumonia affecting different/multiple lobes.

Systemic Immune Disorders
Primary immunodeficiency
Acquired immunodeficiency
Local immune disorders (subtle abnormalities of mucosal defense)
Genetic diseases
Cystic fibrosis
Primary ciliary dyskinesia
Neuromuscular disorders
Central neurologic disease
Peripheral nerve or muscle disease
Conditions causing weakness of expiratory muscles
Airway anomalies
Postinfective or idiopathic bronchiectasis
Multiple complete cartilage rings
Generalized bronchomalacia
Major airway obstruction
Airway compression by enlarged heart or great vessels
Vascular rings and slings
Recurrent aspiration
Severe gastroesophageal reflux
Isolated, late-presenting H-type fistula
Esophageal dysmotility syndromes
Oily medication and nose drops inhalation
Laryngeal cleft
Autoimmune diseases
Pulmonary hemorrhagic syndromes
Allergic bronchopulmonary aspergillosis
Granulomatous disease
Recurrent pulmonary edema (cardiac left-to-right shunting; heart failure)
Drug toxicity

**Table 5:** Characteristics of 113 children with radiologically confirmed recurrent pneumonia from a university hospital in Italy.

Clinical Feature	RP in the Same Area	RP in Different Areas
	(*n* = 28)	(*n* = 85)
Males (%)	43	53
Age at first pneumonia (years) *	1.5 (0.1–6.0)	0.2 (0.1–15.6)
Age at diagnosis of underlying disease (years)	4.7 (1.0–11.8)	6.5 (0.1–37.2)
Underlying diseases (%)		
Middle lobe syndrome	61	0
Localized malacia	11	0
Congenital lung malformation	21	0
Tuberculosis	7	0
Primary immunodeficiency	0	9
Cystic fibrosis	0	5
Primary ciliary dyskinesia	0	51
Severe gastroesophageal reflux	0	2
Esophageal dysmotility	0	5
Pulmonary hemorrhagic syndrome	0	1
Autoimmune disease	0	2
Vascular ring/sling	0	2
Unknown	0	22
Risk factors for RP (%)		
Prematurity	18	9
Atopy	25	27
Tobacco smoke exposure	29	36
Over-crowding	4	15

Values are expressed as median and range; * *p* < 0.001.

**Table 6:** Causes of recurrent pneumonia in the current series, compared with previous studies.

Variable	Current Series	Owayed et al. [5]	Ciftçi et al. [6]	Lodha et al. [7]	Cabezuelo et al. [8]	Hoving et al. [9]	Patria et al. [10]
Number of patients	113	238	71	70	106	62	146
Setting	Tertiary care centre	Tertiary care centre	Tertiary care centre	Tertiary care centre	Tertiary care centre	General hospital	Tertiary care centre
Country	Italy	Canada	Turkey	India	Spain	The Netherlands	Italy
Diagnosis rate (%)	83	92	85	84	87	69	NA
Underlying causes (%)							
Lung/airway disease	26	8	6	9	2	16	30
Immunodeficiency	7	10	10	16	10	16	1
Recurrent aspiration/GER	5	53	18	37	27	26	24
Heart/vessels anomalies	2	9	9	3	29	5	2
Cystic fibrosis	4	0	3	0	0	0	0
Primary ciliary dyskinesia	38	0	0	7 *	0	0	1
Tuberculosis	2	0	3	0	0	0	0

GER, gastroesophageal reflux; * Diagnosis suspected, but not confirmed because of lack of facilities.
